# Collateral Pulmonary Respiration of Van Allen

**Published:** 1983-07

**Authors:** A. M. N. Gardner

**Affiliations:** Department of Surgery, Torbay Hospital


### Collateral Pulmonary Respiration of Van Allen

### A Forgotten Essential Physiological Mechanism for the Clearance of Bronchiolar Blocks

**A. M. N. Gardner, D.M., M.Ch.**
Department of Surgery, Torbay Hospital

Coughing and muco-ciliary clearance are the most important mechanisms in clearing obstructions in the bronchial tree, but they do not themselves explain how the smaller airways can be unblocked for terminal bronchioles have only cuboidal epithelium and no cilia. Some clearance mechanism must exist for otherwise bronchiolar obstruction would inevitably lead to atelectasis in the related alveoli, atelectasis that would be permanent in the absence of alternative paths for alveolar re-inflation, essential to provide air for coughing out the plugs. Although now largely forgotten the alternative path of respiration was described in 1930 by Van Allen.[1]

Van Allen noticed, during experiments on bronchial obstruction in dogs, that after 45 days complete blockage of the medial division of the right lower lobe bronchus there was no associated atelectasis. He subsequently confirmed that, whereas obstruction of a lobar bronchus consistently produces atelectasis, obstruction of a primary division intra-lobar bronchus does not. He concluded that an exchange of air was possible between peripheral parts of the lung lobe.[2]

Further animal experiments showed that collateral respiration was surprisingly efficient, only a small section of the lung being sufficient for aeration of the whole. The efficiency was directly related to the depth of respiration and, as might be expected, the process was impaired by inflammation.[3,9] These observations were confirmed by means of gas analysis by Lindskog and Van Allen.[4]

Collateral respiration was also demonstrated in relation to experimental segmental emphysema produced by valvular bronchial obstruction. Lobar emphysema followed valvular obstruction of lobar bronchi, but no emphysema resulted from this type of obstruction in the primary intra-lobar divisions of the bronchial tree.[5]

Van Allen extended his studies to fresh human lungs at autopsy.[6] Inflation of a primary division bronchus within a lobe caused air to escape from the others. This process started long before full inflation of the lung. He also showed that Indian ink particles could flow collaterally, but not larger (bismuth) particles: observations that fitted with the concept that collateral respiration occurs through the small inter-alveolar 'pores of Kohn'. By study of tuberculous lungs he further extrapolated his animal experiments to man; his careful searches revealed examples of complete bronchial stenosis associated with normally inflated peripheral alveoli.[7,8]

Although Kohn, 1893, was apparently the first to describe inter-alveolar pores,[10] he thought these were pathological rather than normal entities. He described strands of fibrin extending from one alveolus to another through openings in the alveolar walls in lungs affected by acute fibrinous pneumonia and of lungs undergoing 'carnification'. Hauser, 1893, who studied under Kohn, considered such openings were normal and referred to them as the 'pores of Kohn'. Loosli,[10] in 1937, by study of thick stained sections of the lung, showed conclusively that Hauser was correct and the pores were normal rather than pathological structures. He further showed that they were not present at birth but developed later, their development being encouraged by respiratory exertions.

In pathological terms the anatomy of lobar pneumonia is explained by collateral passage of inflammatory fluid through the inter-alveolar pores and in emphysema, where a prominent feature is obstruction or obliteration of peripheral airways, there is evidence that the collateral channels may be important ventilatory pathways.[11,12]

In clinical terms collateral respiration explains how, within a lobe, parenchymal inflation can occur distal to a bronchial block to provide available air for the action of coughing to dislodge the obstruction. Impairment of this mechanism, by shallow breathing in the postoperative period, explains the tendency to postoperative chest problems.

In relation to physiotherapy it explains the effectiveness of deep inspiration and positive end-expiration pressure therapy (PEEP): in its absence these manoeuvres would seem likely merely to move secretions further into the aciliate air passages and so exacerbate respiratory problems.

Van Allen's work seems as important, in relation to respiration, as John Hunter's (also largely forgotten) on the development of collateral circulation to the horn of a deer in Richmond Park after ligation of the external carotoid artery. He noted that pulsation stopped immediately and that the 'hot velvet' at the horn base soon became cold. A week later its temperature was back to normal; the animal was killed and anatomical study showed hypertrophy of arterial branches forming a collateral blood circulation.[13]

### REFERENCES

1. VAN ALLEN, C. M. and ADAMS, W. E. (1930) The mechanism of pulmonary atelectasis. *S.G.O.* **50**, 385–396.
2. VAN ALLEN, C. M. and LINDSKOG, G. E. (1930) Obstructive pulmonary atelectasis. *Arch.Surg.* **21**, 1195–1213.
3. VAN ALLEN, C. M. and JUNG, T. S. (1931) Post-operative atelectasis and collateral respiration. *J.Thoracic Surg.* **1**, 3–14.
4. LINDSKOG, G. E. and VAN ALLEN, C. M. (1931) The aerodynamics of bronchial obstruction. *Arch.Surg.* **24**, 204–230.
5. VAN ALLEN, C. M. (1932) Obstructive pulmonary emphysema and collateral respiration. *S.G.O.* **55**, 303–307.
6. VAN ALLEN, C. M. and LINDSKOG, G. E. (1931) Collateral respiration in the lung. *S.G.O.* **53**, 16–21.
7. VAN ALLEN, C. M., LINDSKOG, G. E. and RICHTER, H. G. (1931) Collateral respiration transfer of air collaterally between pulmonary lobules. *J.Clin.Invest.* **10**, 559–590.
8. LINDSKOG, G. E. and BRADSHAW, H. H. (1934) Collateral respiration, the chemical composition and volume of the collaterally respired gases. *Am.J.Physiol.* **108**, 581–592.
9. VAN ALLEN, C. M. and SOO, Y. C. (1933) Collateral respiration. *J.Clin.Invest.* **12**, 171–179.
10. LOOSLI, C. G. (1937) Inter-alveolar communications in normal and in pathologic mammalian lungs. *Arch.Path.* **24**, 743–776.
11. HOGG, J. C., MACKLEM, P. T. and THURLBECK, W. M. (1969) The resistance of collateral channels in excised human lungs. *J.Clin.Invest.* **48**, 421–431.
12. TERRY, P. B., TRAYSTMAN, R. J., NEWBALL, H. H., BATRA, G. and MENKES, H. A. (1978) Collateral ventilation in man. *New England J.Med.* **298**, 10–15.
13. OWEN, R. (1897) In Descriptive Catalogue of the Physiological Series Hunterian Museum. Royal College of Surgeons (1970), Vol. 1, p. 8.

